# Post Circumcision Intraperitoneal Rupture of the Urinary Bladder: A Rare Complication

**DOI:** 10.1055/s-0039-1700986

**Published:** 2020-03-06

**Authors:** Sherif Abdelmaksoud, Mohammed Albishbishy, Mostafa Elayyouti, Mohamed Zohiri, Adham Elsaied

**Affiliations:** 1 Department of Paediatric Surgery, Mansoura University Children's Hospital, Mansoura, Egypt, Egypt

**Keywords:** circumcision, intraperitoneal, bladder rupture, rare complication

## Abstract

Circumcision is one of the most common pediatric surgical procedures performed all over the world and especially in Arab and Islamic countries. Many complications have been documented following this maneuver. We report on a rare case of intraperitoneal bladder rupture in a 7-day-old baby who was circumcised on his second day using the guillotine method. He presented to us with gangrene of the tip of the penis and a failure to void urine associated with progressive abdominal distension. Ultrasound revealed severe ascites. Aspiration and analysis confirmed the fluid to be urine. Ascending cystourethrogram was performed revealing a perforation of the posterior bladder wall near the trigone. Exploration was performed and repair done. Postoperative course was uneventful.

## Introduction

Male circumcision is one of the commonest surgical procedures practiced in children worldwide. According to several studies, almost a third of the world male population is circumcised. 1 2 A complication rate of up to 16% has been reported. 1 Bladder rupture is a very rare and life-threatening complication of such a procedure. In a review of literature, only three such cases were reported with this being the fourth. 3 4 5 We would like to report on a 7-day-old patient that suffered from an intraperitoneal bladder rupture following neonatal male circumcision.

## Case Report

Our patient presented to our emergency department on the seventh day of life with severe abdominal distension and vomiting. A thorough history revealed that the baby was circumcised on the second day of life following an uncomplicated cesarian section delivery. The exact mode of circumcision was unknown to the parents. After this the baby was found to be fussy and constantly crying and was no longer tolerating his feeds. He then started to become lethargic and show progressive abdominal distension. The baby was transferred to a neonatal unit with a provisional diagnosis of neonatal sepsis. Three days later he was referred to our center to assess and manage his ongoing abdominal distension.

**Fig. 1:**
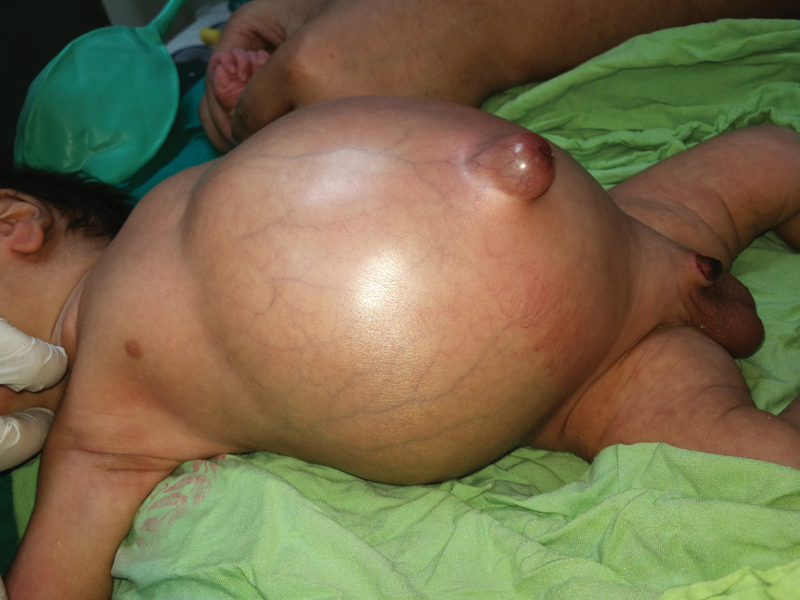
Clinical picture of the baby at first presentation. Notice the gross distended abdomen, dilated abdominal veins, mild erythema of the abdominal wall and dry gangrene of the distal glans of the penis.

General examination revealed an elevated temperature of 38°C, pulse of 155, and a blood pressure of 70/38. The abdomen was diffusely enlarged with prominent dilated veins, a large protuberant umbilical hernia, and mild erythema. There was no tenderness on palpation. It was dull all over on percussion with the evidence of a transmitted thrill. The baby was passing stool normally and there were no bilious aspirates when an nasogastric tube was placed. Careful examination of the site of circumcision revealed that the glans penis was ischemic with signs of inflammation at the shaft ( Fig. 1 ). Laboratory investigations revealed an elevated serum creatinine level of 2.3 mg/dL, C-reactive protein of 96 mg/L, hyperkalemia (5.7 mMol/L), hyponatremia (122 mMol/L), and an elevated leucocyte count of 15,000/mm ^3^ .

The baby was immediately admitted and resuscitation was performed. An abdominal X-ray failed to show any free air in the abdomen and a concomitant abdominal ultrasound showed marked free fluid with internal echoes and bilateral moderate hydronephrosis. A provisional diagnosis of intraperitoneal bladder rupture was made based on this data and a conservative approach was decided. An abdominal drain was placed in the operating room under general anesthesia yielding >500ccs of yellowish turbid fluid. A urinary catheter was placed to drain the bladder; however, catheterization was difficult due to involvement of the distal part of the penile urethra.

**Fig. 2:**
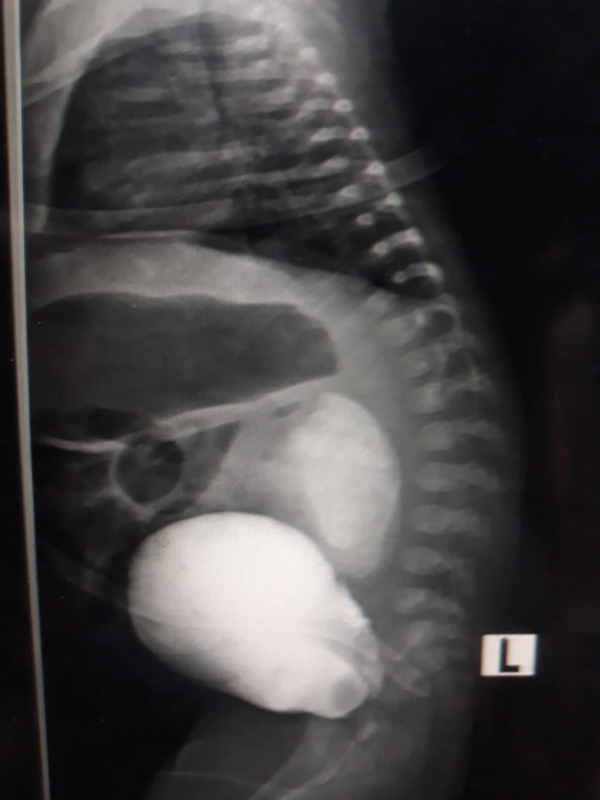
Ascending cytogram (lateral view) showing an intraperitoneal leakage of contrast from the dome of the bladder posteriorly close to the trigone.

**Fig. 3:**
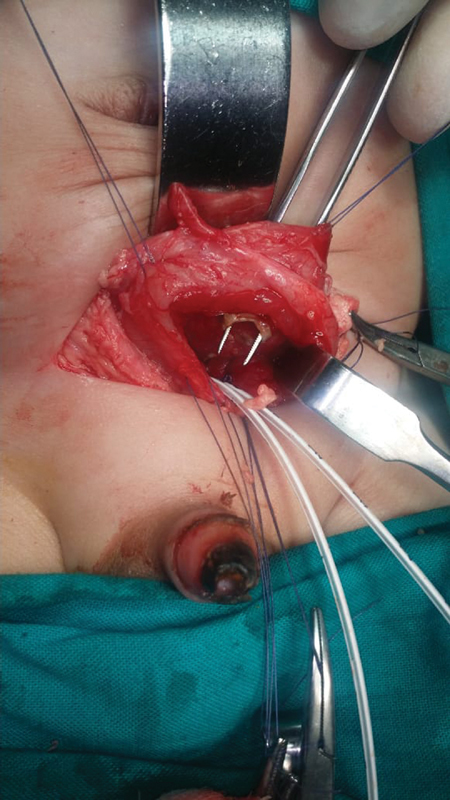
Intraoperative view. The bladder is bivalved (opened), the ureters are cannulated and the perforation is evident.

The baby remained under conservation for 2 days after which a retrograde cystogram was performed through the catheter revealing leakage of contrast from the bladder near the trigone into the peritoneum ( Fig. 2 ). An open exploration was decided upon through a Pfannenstiel incision. Due to the close proximity of the perforation to the ureters, we decided to approach from within the bladder to allow us to cannulate the ureters and avoid injuring them during the repair ( Fig. 3 ).

Two pathological perforations were appreciated near the trigone between the ureteric orifices. These were debrided and closed in two layers with 4/0 polygalactine suture. The bladder was then closed over a suprapubic self-retaining catheter. An abdominal toilet was done, a drain was placed, and the incision was closed. A clear line of demarcation was visible at the time of surgery between the gangrenous and healthy penile tissue as almost a week had passed since the initial injury. This prompted us to perform debridement of the dead tissue to avoid sending the patient for a second general anesthetic.

The baby was transferred to the surgical neonatal intensive care unit where he remained on broad-spectrum antibiotics for 3 days. Oral feeds were commenced on day 2. On day 5 postoperatively, a contrast study was done through the suprapubic catheter revealing absence of any leakage. The suprapubic catheter was then clamped. When the baby was voiding normally the catheter was removed. The baby was discharged on the seventh postoperative day.

## Discussion

Neonatal bladder rupture is a rare problem that has been reported secondary to several different pathologies. The most common of these is urinary tract obstruction due to a posterior urethral valve or anterior urethral valve. 6 7 It has also been reported on as a possible complication of umbilical catheterization 8 and severe neonatal urinary tract infection (UTI). 9 Only three cases of bladder rupture following circumcision have been reported in literature till today. Two of these were due to circumcision with a Plastibell device where the urinary tract had become obstructed due to migration and trapping of the inner perpetual skin beneath it. 3 4 The third case was in a 2-year-old child and had occurred when deep hemostasis sutures were taken following circumcision to control bleeding and the surgeon had inadvertently damaged the distal subcoronal urethral causing urinary tract obstruction. 5

In our patient, the situation was a little different. The circumcision was performed by a traditional practitioner employing mostly a bone cutter. Our hypothesis is that the distal end of the glans had become caught in the device causing a crush injury to it and the most distal part of the urethra and this wasn't managed correctly unfortunately. This caused an acute urinary retention that was missed on initial examination and ultimately led to pressure build up and bladder rupture.

Our initial management was conservative through catheterization of the bladder per urethra and drainage of the urinary ascites after proper patient resuscitation. The decision was made for exploration due to a worsening general condition and the retrograde cytogram that demonstrated an intraperitoneal leak. Both modalities of treatment have been proposed to manage intraperitoneal bladder rupture; however, the inclination is usually toward a formal surgical repair especially when there is a deterioration of the patient's general condition. Our management was in accordance with published guidelines from the consensus on genitourinary trauma which underlined that neonatal bladder trauma usually requires surgical management. 10 In addition, published case reports on similar conditions ultimately resorted to surgical intervention to as a course of management. 2 3 4 5 6 7 8 9

Most authors report that an open approach but some have advocated for a laparoscopic approach to repair bladder ruptures. 6 In any case, the repair is performed from outside the bladder. In our patient, we opted to bivalve the bladder and perform the repair from inside due to the close proximity of the perforation to the ureters and fear of the repair leading to ureteric obstruction. This approach allowed us to identify and cannulate both ureters in addition to performing a mucosal and seromuscular repair of the bladder.

Long-term monitoring of patients where suturing in done near the trigone is vital as such patients are at a higher risk of long-term bladder dysfunction. 11 We were unable to do so in this patient unfortunately as he was lost to follow-up.

Neonatal circumcision remains a controversial subject to this day. Many studies have been designed to assess the advantages and disadvantages of this practice. Recently the role of neonatal male circumcision in the reduction in the risk of UTIs has been emphasized. 12 Furthermore, it has been shown that neonatal circumcision if performed under the appropriate controlled conditions has a much lower risk of short-term complications than if performed at an older age. The risk of additional surgery is in fact almost 1% if circumcision was initially performed after the neonatal period. 13

However, this statement isn't supported by many. The relationship between circumcision and the development of UTIs according to several authors is only established in those with a predisposition to the condition such as those boys with PUJ (pelviureteric junction) obstruction, high-grade vesicoureteral reflux, and hydronephrosis. 14 This is why they feel that routine nontherapeutic practice of circumcision should be restricted to those subgroups of patients. To add to this point, it was found that the risk rate for the development of meatal stenosis and other urethral stricture diseases was higher in religious and cultural ethnicities that practiced nontherapeutic circumcision. This greatly adds to the health service burden. 15

It is our message that although circumcision is performed routinely in many cultures, it can still lead to catastrophic and life-threatening problems adding a considerable cost to the already taxed health services especially in low- and middle-income countries. It cannot be stressed enough that this should only be performed by a trained professional under safe, sterile circumstances and where a proper follow-up can be provided to detect and manage any complications as early as possible should they occur.

## Notes

New Insights & the Importance for the Pediatric Surgeon Circumcision is one of the most practiced pediatric surgery procedures. This is the fourth reported case of bladder rupture as a complication to this procedure worldwide.
